# The use of mixed reality technology for the objective assessment of clinical skills: a validation study

**DOI:** 10.1186/s12909-022-03701-3

**Published:** 2022-08-23

**Authors:** Iona Minty, Jason Lawson, Payal Guha, Xun Luo, Rukhnoor Malik, Raminta Cerneviciute, James Kinross, Guy Martin

**Affiliations:** Department of Surgery and Cancer, Imperial College London, St Mary’s Hospital, 10th Floor QEQM Building, London, W2 1NY UK

**Keywords:** Medical education, Assessment, Mixed reality technology, Innovation

## Abstract

**Background:**

Mixed Reality technology may provide many advantages over traditional teaching methods. Despite its potential, the technology has yet to be used for the formal assessment of clinical competency. This study sought to collect validity evidence and assess the feasibility of using the HoloLens 2 mixed reality headset for the conduct and augmentation of Objective Structured Clinical Examinations (OSCEs).

**Methods:**

A prospective cohort study was conducted to compare the assessment of undergraduate medical students undertaking OSCEs via HoloLens 2 live (HLL) and recorded (HLR), and gold-standard in-person (IP) methods. An augmented mixed reality scenario was also assessed.

**Results:**

Thirteen undergraduate participants completed a total of 65 OSCE stations. Overall inter-modality correlation was 0.81 (*p* = 0.01), 0.98 (*p* = 0.01) and 0.82 (*p* = 0.01) for IP vs. HLL, HLL vs. HLR and IP vs. HLR respectively. Skill based correlations for IP vs. HLR were assessed for history taking (0.82, *p* = 0.01), clinical examination (0.81, *p* = 0.01), procedural (0.88, *p* = 0.01) and clinical skills (0.92, *p* = 0.01), and assessment of a virtual mixed reality patient (0.74, *p* = 0.01). The HoloLens device was deemed to be usable and practical (Standard Usability Scale (SUS) score = 51.5), and the technology was thought to deliver greater flexibility and convenience, and have the potential to expand and enhance assessment opportunities.

**Conclusions:**

HoloLens 2 is comparable to traditional in-person examination of undergraduate medical students for both live and recorded assessments, and therefore is a valid and robust method for objectively assessing performance. The technology is in its infancy, and users need to develop confidence in its usability and reliability as an assessment tool. However, the potential to integrate additional functionality including holographic content, automated tracking and data analysis, and to facilitate remote assessment may allow the technology to enhance, expand and standardise examinations across a range of educational contexts.

**Supplementary Information:**

The online version contains supplementary material available at 10.1186/s12909-022-03701-3.

## Background

Innovative mixed reality (MR) technologies have the potential to transform the delivery of medical education [1], and may confer some advantages over traditional teaching methods by merging real and virtual worlds [2, 3]. The technology has the potential to help tackle many of the challenges currently faced in the delivery of high-quality medical education globally including quality, consistency, accessibility and cost [4, 5].

The HoloLens 2 (HL2) is a commercially available MR headset produced by Microsoft (Microsoft Corporation, Redmond, WA, USA) that allows for remote first-person visualisation, multi-directional audio and visual communication, and the integration and manipulation of interactive 3-dimensional (3D) holographic content into real-world scenarios [6]. The device has been deployed into a range of clinical settings including ward-based care, pre-operative planning, and intra-operative visualisation [7, 8]. The technology has also been successfully integrated into medical schools’ curricula, principally to support the delivery of anatomy teaching through a range of commercial and bespoke applications [9–11]. More recent developments have allowed the development of integrated clinical skills teaching sessions [12, 13] in which immersive multi-sensory (audio, visual, tactile) content can be created to imitate real-world scenarios [14]. Despite rapid progress in the creation educational content, there is only limited experience in its use for objective assessment or examination.

The HL2 device may facilitate remote assessment both in real-time and via recorded content. This approach may not only reduce cost and improve access to qualified assessors, but may also facilitate assessments to be taken out of the abstract structured environment of formal examinations and into real-life opportunistic clinical interactions. In addition, the use of interactive MR content provides opportunities to augment the assessment process through the use of holographic assets, or the use of interactive instructional material and clinical information [15, 16]. Despite the clear potential to augment and enhance approaches to assessment, no institution has yet evidenced use of the technology in formal examinations [17], nor has it been validated as an effective and robust assessment tool.

## Methods

The study sought to examine the feasibility and validity of using the HL2 MR headset for objective assessment and augmentation of Objective Structured Clinical Examinations (OSCEs) across a range of core undergraduate clinical competencies.

### Participants

Thirteen undergraduate medical student participants were recruited. All were at, or above the level of proficiency required to complete the study, and none had prior experience of using a HL2 device. The study received institutional educational ethical approval (EERP2021-055) and written informed consent was obtained from all participants.

### Study design

**Fig. 1:**
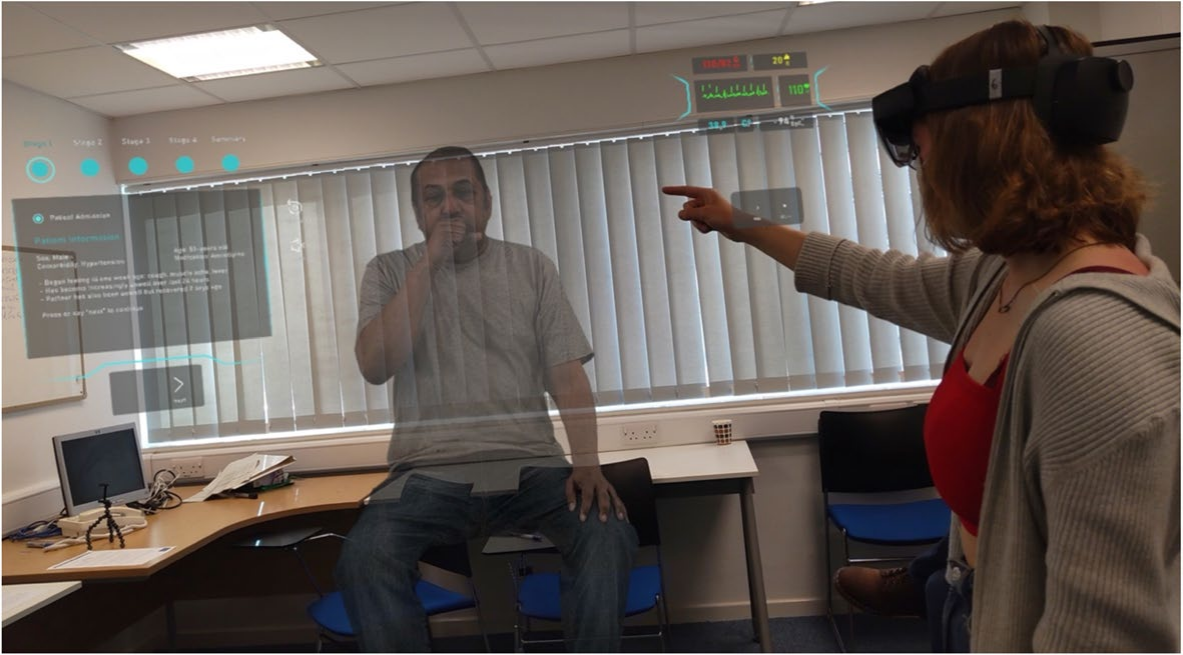
3rd person view of the HoloPatient [16] being deployed as a simulated patient for the purposes of examining clinical competencies in assessing a critically unwell patient

This prospective cohort study was conducted to collect validity evidence for the use of MR technology as a tool for the objective assessment of undergraduate clinical competencies by comparing it to the current gold-standard in-person method of examination. Study accrual was based on convenience sampling comparable to similar studies as no power calculation was practicable due to the novel data being assessed. Participants undertook an OSCE examination consisting of five stations representative of their assessed curriculum. Each station examined a different domain of mandatory core clinical competencies encompassing clinical examination, history, and procedural and skills-based assessment, and utilised actors and synthetic benchtop models. A final station introduced a virtual simulated COVID-19 patient provided publicly for free by GigXR (GigXR Inc, Venice, CA, USA) that created an immersive learning environment simulating a deteriorating patient [16] to examine the potential for MR technology to transform or augment the assessment process (Fig. 1).

Participants undertook all five stations on rotation. Ten minutes were provided to complete each station with one-minute intervals between, mimicking the local standardised clinical OSCE examination format. A HL2 device was worn by each student whilst completing the study following a period of standardised training on how to wear and operate the device. Each station was assessed via three modalities: the current gold-standard in person assessment by a trained examiner in the room (IP), virtually in real-time using the HL2 device linked to a trained examiner (HLL), via the Microsoft Remote Assist software platform (Microsoft Corporation, Redmond, WA, USA) and finally via a recording of the scenario obtained from the HL2 device (HLR). All examiners were qualified doctors with proficiency to assess the core competencies examined as part of the study. Examiners rated all three arms of the study and intra-modality variability was assessed to ensure consistency of performance. Indicative mark schemes for each station are provided in Additional file 1. Participant feedback data were also collected, and usability of the device assessed vis the Standard Usability Scale (SUS) [18]. The primary outcome was the inter-modality correlation and inter-rater variability with the current gold-standard in-person method of assessment. The study was conducted in accordance with all relevant guidelines, regulations and the principles of the Declaration of Helsinki.

### Statistical analysis

Standard descriptive statistics were employed. Normality of data were assessed via Shapiro–Wilk tests and two Tailed Pearson’s and Spearman Rank Correlation Coefficients were calculated. Inter-modality variability was examined by Cronbach Alpha Intra Class Coefficient. Correlations were classified according to the correlation coefficient [19]. All data were collated and analysed in Microsoft Excel (V16.48, Microsoft Corporation) and IBM SPSS (V27, IBM Corporation), and charts produced in Prism 9 [Version 9.1.0 (216)].

## Results

### Assessment of performance

**Fig. 2:**
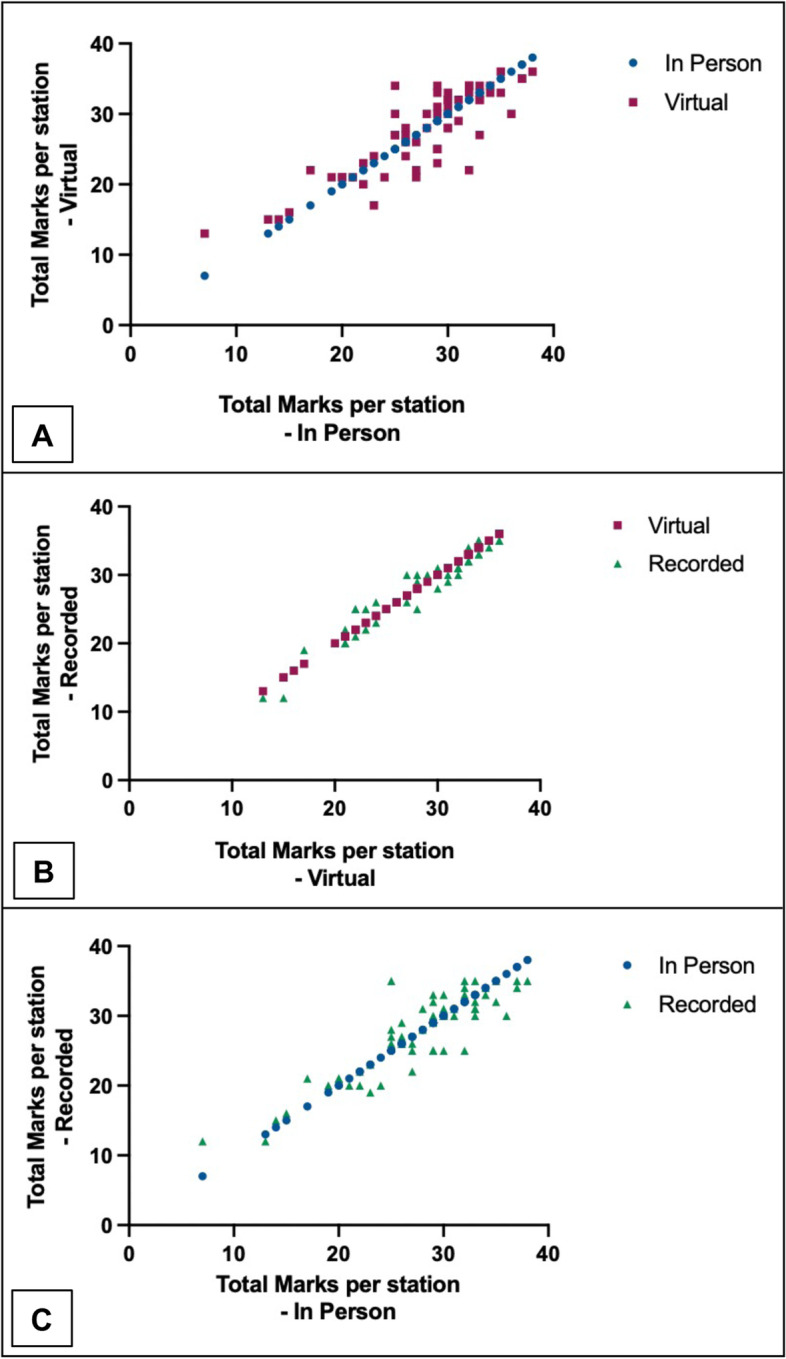
Combined inter-modality correlations for each assessment modality across all five clinical domains. **A** IP vs. HLL 0.81 (*p* =  < 0.01). **B** HLL vs. HLR 0.98 (*p* =  < 0.01). **C** IP vs. HLR 0.82 (*p* =  < 0.01). IP = in person assessment, HLL = HoloLens live assessment, HLR = HoloLens recorded assessment

**Fig. 3:**
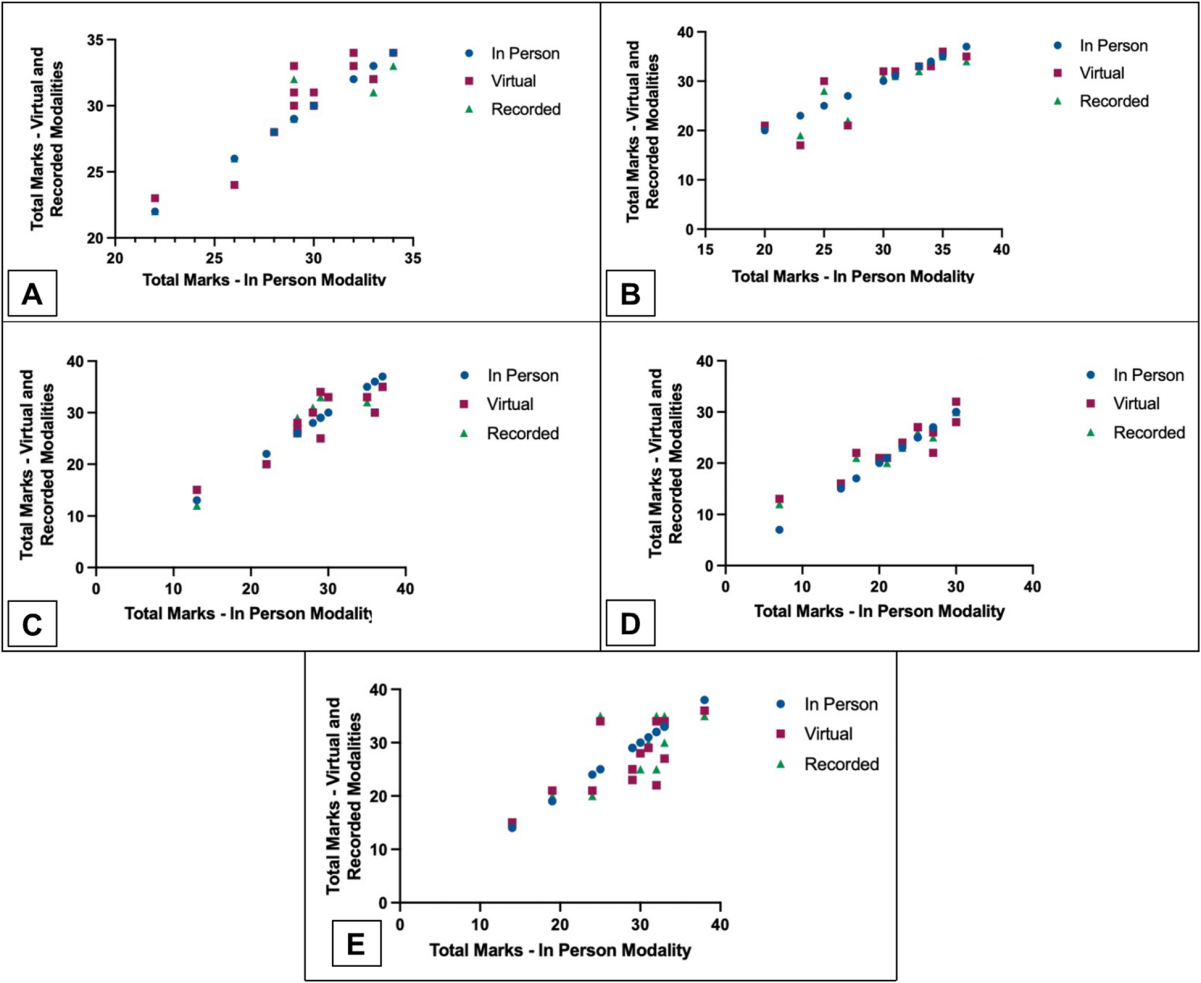
Inter-modality correlations for HL vs. IP assessments across each of the five clinical competencies assessed. **A** Clinical history 0.89 (*p* =  < 0.01). **B** Clinical examination 0.97 (*p* =  < 0.01). **C** Procedural skill 0.94 (*p* =  < 0.01). **D** Basic clinical skills 0.95 (*p* =  < 0.01). **E** Simulated patient assessment 0.72 (*p* =  < 0.01). HL = HoloLens live + HoloLens recorded assessments. IP = in person assessment

Overall combined inter-modality correlations were 0.81 (*p* =  < 0.01), 0.98 (*p* =  < 0.01) and 0.82 (*p* =  < 0.01) for IP vs. HLL, HLL vs. HLR and IP vs. HLR respectively (Fig. 2). Overall combined correlations were 0.97 (*p* =  < 0.01), 0.89 (*p* =  < 0.01), 0.94 (*p* =  < 0.01) and 0.95 (*p* =  < 0.01) for clinical examination, history, procedural and skills-based assessments respectively (Fig. 3). The correlation co-efficient for each individual skill type and assessment modalities are provided in Table 1.

**Table 1:** Three-way intramodality analysis including the Correlation Co-efficient (*r*) and significance and the Intra Class Coefficient (ICC) measure of inter-participant variability for all five clinical domains assessed

Modalities	Two-tailed Correlation Co-efficient (*r*)	Significance	Intra Class Coefficient (ICC)
*In Person (IP) vs. HoloLens Live (HLL)*			
Combined Score	0.810 (*p* < 0.01)	Strong	0.920 (0.865–0.952)
Clinical History	0.896 (*p* < 0.01)	Strong	0.944 (0.817–0.983)
Clinical Examination	0.942 (*p* < 0.01)	Strong	0.916 (0.663–0.979)
Procedural Skill	0.814 (*p* < 0.01)	Strong	0.931 (0.761–0.980)
Clinical Skill	0.871 (*p* < 0.01)	Strong	0.938 (0.785–0.982)
HoloPatient	0.708 (*p* < 0.01)	Strong	0.843 (0.485–0.952)
*HoloLens Live (HLL) vs. HoloLens Recorded (HLR)*			
Combined Score	0.978 (*p* < 0.01)	Strong	0.988 (0.980–0.993)
Clinical History	0.962 (*p* < 0.01)	Strong	0.979 (0.931–0.987)
Clinical Examination	0.988 (*p* < 0.01)	Strong	0.990 (0.960–0.998)
Procedural Skill	0.993 (*p* < 0.01)	Strong	0.993 (0.975–0.998)
Clinical Skill	0.973 (*p* < 0.01)	Strong	0.982 (0.939–0.998)
HoloPatient	0.959 (*p* < 0.01)	Strong	0.983 (0.943–0.995)
*In Person (IP) vs. HoloLens Recorded (HLR)*			
Combined Score	0.823 (*p* < 0.01)	Strong	0.939 (0.898–0.964)
Clinical History	0.810 (*p* < 0.01)	Strong	0.951 (0.839–0.985)
Clinical Examination	0.951 (*p* < 0.01)	Strong	0.951 (0.803–0.988)
Procedural Skill	0.880 (*p* < 0.01)	Strong	0.936 (0.777–0.982)
Clinical Skill	0.926 (*p* < 0.01)	Strong	0.971 (0.901–0.992)
HoloPatient	0.743 (*p* < 0.01)	Strong	0.867 (0.565–0.959)

Utilising MR content to augment traditional approaches to assessment also has promise. Participants demonstrated comparable overall performance in the holographic patient scenario compared to real patient scenarios with mean scores of 28.4 and 29.8 respectively (*p* = 0.42), and once again correlations across all three assessment modalities with the holographic patient were strong: IP vs. HLL (0.71, *p* =  < 0.01), HLL vs. HLR (0.96, *p* =  < 0.01) and IP vs. HLR (0.74, *p* =  < 0.01).

The HL2 device is also a reliable and consistent assessment modality when used by a range of assessors with ICC scores consistently > 0.9 for all live patient scenarios and > 0.8 for the holographic patient scenario, indicating excellent reliability across all domains (Table 1).

### Participant feedback and usability

100% (13 participants, 8 assessors) agreed or strongly agreed that the integration of MR technology has the potential to enhance the assessment experience. *“I see a lot of potential for the future implementation of the HoloLens for practical exams such as PACES and OSCEs.”* (Student). 12/13 (92.3%) of student participants and 5/8 (62.5%) of assessors stated that the first-person view allowed for better visualisation of student performance compared to in-person assessment. 11/13 (85.6%) of students and 8/8 (100%) of assessors agreed or strongly agreed that they would like to see the technology incorporated into future assessments. 12/13 (92.3%) students agreed or strongly that recording their performance could be useful for self-directed learning and future development. 8/8 (100%) of assessors agreed or strongly agreed that scoring a recording of a students’ performance instead of in person allows for greater flexibility, convenience and accessibility. *“HoloLens 2 allowed greater flexibility both in real-time and also for re-watching recordings at a later date.”* (Assessor).

Feedback on the technology was not universally positive, however. 6/13 (46.2%) of participants reported general discomfort or difficulty concentrating with the HL2 device, although none reported symptoms of headache, fatigue, nausea or eye strain. 12/13 (92.3%) of students and 2/8 (25.0%) of assessors reported that the device potentially interfered with their ability to carry out the required task. This was principally due to unfamiliarity with the technology and software usability despite a period of standardised training, rather than the physical impact of the device; issues which will likely dimmish as familiarity with the technology grows. *“HoloLens 2 looks to be overall a very promising and novel method of teaching and assessing, one that certainly has a few early teething problems with implementation, but I am confident once over the learning curve and made more widely accessible, will prove to be a very useful tool piloted in medical education but with possible widespread applications across multiple industries.”* (Assessor).

Overall, the HL2 technology was reported to be moderately usable, with an SUS score of 51.5 [18]. The highest scoring domains were ease of use, function integration and confidence in using technology. The lowest scoring domains were overall complexity and the need to learn a lot of things to get going with the technology, and the need for specialist technical support to implement the technology in day-to-day practice.

## Discussion

HoloLens 2 appears to be comparable to in-person examination of undergraduate medical students for both live and recorded assessment, and therefore is a valid and robust method for objectively assessing performance across a full range of core competencies including clinical examination, history taking, and clinical and procedural skills. The use of MR content, in this case a virtual patient, has been shown to have the potential to augment the assessment process with students and assessors performing comparably to clinical interactions with real simulated patients.

MR technology has the potential to unlock a wide range of novel assessment techniques and opportunities. It may facilitate the summative appraisal of tasks conducted in representative real-life contexts through the assessment of opportunistic clinical interactions and skills. The ability to facilitate the assessment of infrequent, or challenging competencies such as breaking bad news, or rare clinical presentations and procedures provides opportunities for increasing the scope of formal assessment. The use of virtual logbooks may also facilitate the provision of greater, and higher quality supervision and mentoring from educators who cannot be present to undertake assessment or feedback in real-time. In addition, it may support a more robust approach to quality assurance, and allow for greater transparency in decision making, particularly when there may be disputes regarding performance or ability. Given that most medical students are dissatisfied with the feedback they receive, introducing new technologies that support more robust, transparent, and engaging assessment processes should be championed [20]. The potential for the technology to deliver an online repository of student performance across a range of settings throughout their training has huge potential and implications for medical education more widely.

MR technology such as the HL2 device provides seamless integration with cloud services, and the ability to deploy intuitive software platforms that can integrate holographic content and additional functionality that are supported by a range of sensors and hardware on the device. Whilst in its infancy, the use of holographic content to augment the assessment process has been shown to be robust and valid, and broadly comparable to the use of live patients. Despite showing potential, the use of holographic material only supports the assessment of specific parts of clinical interactions. It remains to be demonstrated that a holographic patient, or other MR content can fully replicate more complex and nuanced aspects such as non-verbal cues or subtleties of language, communication and inter-personal interaction. In addition, the substantial gap in the technology is its inability to replicate key multi-sensory parts of basic physical examination such as palpation or auscultation. Given these limitations, whilst there may be a role for MR content to augment the assessment process, the technology is too immature currently to completely replace real or actor patients.

Despite showing promise there remain several barriers to its widespread adoption and successful scaling of the technology. Key to this are inherent limitations found with current generation of hardware and software. The battery life of the device limits the time it can be used, with often only around 60 min of continuous use achieved, potentially limiting its applicability to longer forms of assessment. In addition, there are limitations to the number of applications that can be run at any point in time without the device crashing, which may restrict the ability to augment the assessment process to the technologies full potential due to limitations with current off-the-shelf software applications. The significant financial resources required—$3,500 per device, with individual software licences in addition—may act as a barrier to widespread adoption but may conversely also be seen as an effective way to minimise the cost of exams by negating the need to bring together examiners, students, and patients in traditional structured formats. Finally, there is a learning curve to the technology. For example, the HL2 device is principally controlled by hand gestures, with supination and tapping of the wrist returning to user to the home screen; a movement which closely mimics the action of putting on gloves during a clinical scenario. If the device it to be used more widely, then solutions or mitigations to these issues must be developed.

One out of the five assessors reported concerns that the HL2 interfered with their ability to assess the student compared to being present in the room, and most students were also concerned that the device my hinder their performance. These concerns were not however borne out, with students and assessors displaying consistent performance across all modalities. It has been demonstrated that MR headsets have no impact on cognitive function [21], and this study would suggest the technology also does not impact task performance despite a large minority of students reporting discomfort or difficulty concentrating whilst using the device. The understandable perception that wearing such devices negatively impacts performance, and potential for physical discomfort whilst wearing the device is a clear barrier to implementation. Importantly, despite this the study has indicated that the use of holographic content to replace or augment traditional aspects of examinations whilst in its infancy is robust. Participants displayed broadly comparable performances compared to traditionally structured exams, consistent with previous data [22].

The additional limitations of this study, primarily that of convenience sampling from a single institution and a small study size leave the risk of type II error and bias impacting the results. Any new technology, and the assessment of it, will also be influenced by technology bias. There is a need to minimise these potential confounders and to evaluate the technology at scale across a range of contexts, and not just in a digitally advanced and engaged institution as in this study to determine its wider applicability. Much of the study utilised the basic core functions of the HL2 device, namely the head-mounted camera and microphone. An important additional evaluation that was not undertaken would be to compare performance of students and assessors when using the HL2 device compared to fixed cameras within the examination room. This would not only deliver further evidence for the potential impact of a head-mounted device on performance but would also provide further context to demonstrate the added value of holographic content and the other additional HL2 device functionality.

HL2 has previously been integrated into medical school curricula [2] and can be effective tool for delivering engaging teaching sessions using realistic holographic models [4, 9] of sufficient detail to replicate traditional approaches using cadaveric models [10].The use of a simulated holographic patient in this study provides insight into the potential for expanding the use of realistic holographic content beyond just teaching, and into simulation and assessment. The ability to replicate and manipulate physical signs and observational data in real-time provides the opportunity to deliver a far wider range of dynamic examination scenarios. The addition of conversational interfaces and speech recognition capabilities that can respond and interact has the potential to be transformative and support a paradigm shift in how students are examined and assessed [20, 23, 24]. Integration of the additional functionality available on the HL2 device provides the opportunity introduce entirely novel approaches to assessment. For example, integrated eye-tracking can be used for novel gaze-based techniques [25] to assess gaze, attentiveness and students’ focused engagement with tasks. Integrated hand-tracking may be useful in the assessment of technical skills by allowing entirely new domains of performance and progression to be captured [26, 27] through measuring precision and economy of movement that has been shown to be an objective measure of technical skill [28, 29].

## Conclusion

HoloLens 2 is comparable to traditional in-person examination and appears to be a valid and robust method for objectively assessing performance across a variety of core clinical competencies. The technology is in its infancy and still requires considerable development, and users need to gain confidence in its usability and reliability as an assessment tool. However, the potential to integrate additional functionality and to facilitate remote or ad-hoc assessments may allow the technology to enhance, expand and standardise examinations across a range of educational settings.

## Supplementary Information

**Additional file 1:** **Table S1.** History Station (Leg Pain) Mark Scheme. **Table S2.** Examination Station (Lower Limb) Mark Scheme. **Table S3.** Procedure Station (Peripheral Cannulation) Mark Scheme. **Table S4.** Skills Station (Simple Suturing) Mark Scheme. **Table S5.** History and Assessment (Simulated Patient) Mark Scheme.

## Data Availability

The datasets generated and/or analysed during the current study are not publicly available as they contain potentially identifiable participant information. They are available from the corresponding author on reasonable request.

## References

[1] Pennefather P, Krebs C (2019). Exploring the Role of XR in Visualisations for Use in Medical Education. Adv Exp Med Biol.

[2] Bogomolova K, Sam A, Misky A (2021). Development of a Virtual Three-Dimensional Assessment Scenario for Anatomical Education. Anat Sci Educ.

[3] Gerup J, Soerensen C, Dieckmann P (2020). Augmented reality and mixed reality for healthcare education beyond surgery: an integrative review. Int J Med Educ.

[4] Wish-Baratz S, Crofton A, Gutierrez J, Henninger E, Griswold M (2020). Assessment of Mixed-Reality Technology Use in Remote Online Anatomy Education. JAMA Netw Open.

[5] Ruthberg J, Tingle G, Tan L (2020). Mixed reality as a time-efficient alternative to cadaveric dissection. Med Teach.

[6] Martin G, Koizia L, Kooner A (2020). Use of the HoloLens2 Mixed Reality Headset for Protecting Health Care Workers During the COVID-19 Pandemic: Prospective, Observational Evaluation. J Med Internet Res.

[7] Mitsuno D, Ueda K, Hirota Y, Ogino M (2019). Effective Application of Mixed Reality Device HoloLens. Plast Reconstr Surg.

[8] Tepper O, Rudy H, Lefkowitz A (2017). Mixed Reality with HoloLens. Plast Reconstr Surg.

[9] Kumar N, Pandey S, Rahman E (2021). A Novel Three-Dimensional Interactive Virtual Face to Facilitate Facial Anatomy Teaching Using Microsoft HoloLens. Aesthetic Plast Surg.

[10] Maniam P, Schnell P, Dan L (2019). Exploration of temporal bone anatomy using mixed reality (HoloLens): development of a mixed reality anatomy teaching resource prototype. J Vis Commun Med.

[11] Robinson B, Mitchell T, Brenseke B (2020). Evaluating the Use of Mixed Reality to Teach Gross and Microscopic Respiratory Anatomy. Med Sci Educ.

[12] Schoeb D, Schwarz J, Hein S (2020). Mixed reality for teaching catheter placement to medical students: a randomized single-blinded, prospective trial. BMC Med Educ.

[13] Muangpoon T, Haghighi Osgouei R, Escobar-Castillejos D, Kontovounisios C, Bello F (2020). Augmented Reality System for Digital Rectal Examination Training and Assessment: System Validation. J Med Internet Res.

[14] Balian S, McGovern S, Abella B, Blewer A, Leary M (2019). Feasibility of an augmented reality cardiopulmonary resuscitation training system for health care providers. Heliyon.

[15] Microsoft Corporation 2021. Microsoft Mixed Reality / AR Guides | Microsoft Dynamics 365. https://dynamics.microsoft.com/en-us/mixed-reality/guides. Accessed 1 May 2021.

[16] GIGXR 2021. HoloPatient Immersive Pathology Exploraton. https://www.gigxr.com/applications/holopatient. Accessed 1 May 2021.

[17] Mackay M 2019. Imperial and Leiden University collaborate on world-leading AR assessment. https://www.imperial.ac.uk/news/191655/imperial-leiden-university-collaborate-world-leading-ar/. Accessed 5 May 2021.

[18] Usability.gov 2021. System Usability Scale (SUS). https://www.usability.gov/how-to-and-tools/methods/system-usability-scale.html. Accessed 9 May 2021.

[19] Cronk B (2021). How To Use SPSS® A Step-By-Step Guide To Analysis And Interpretation.

[20] Brits H, Bezuidenhout J, van der Merwe LJ, Joubert G (2020). Students' voices: assessment in undergraduate clinical medicine. Pan Afr Med J.

[21] Cometti C, Païzis C, Casteleira A, Pons G, Babault N (2018). Effects of mixed reality head-mounted glasses during 90 minutes of mental and manual tasks on cognitive and physiological functions. PeerJ.

[22] Castro-Yuste C, García-Cabanillas M, Rodríguez-Cornejo M, Carnicer-Fuentes C, Paloma-Castro O, Moreno-Corral L (2018). A Student Assessment Tool for Standardized Patient Simulations (SAT-SPS): Psychometric analysis. Nurse Educ Today.

[23] Chung A, Griffin A, Selezneva D, Gotz D (2018). Health and Fitness Apps for Hands-Free Voice-Activated Assistants: Content Analysis. JMIR Mhealth Uhealth.

[24] Isbitski D, Fishman E, Rowe S (2021). Connecting With Patients: The Rapid Rise of Voice Right Now. J Am Coll Radiol.

[25] Kapp S, Barz M, Mukhametov S, Sonntag D, Kuhn J (2021). ARETT: Augmented Reality Eye Tracking Toolkit for Head Mounted Displays. Sensors.

[26] Lu S, Sanchez Perdomo Y, Jiang X, Zheng B (2020). Integrating Eye-Tracking to Augmented Reality System for Surgical Training. J Med Syst.

[27] Frantz T, Jansen B, Duerinck J, Vandemeulebroucke J (2018). Augmenting Microsoft's HoloLens with vuforia tracking for neuronavigation. Healthc Technol Lett.

[28] Yamaguchi S, Yoshida D, Kenmotsu H (2010). Objective assessment of laparoscopic suturing skills using a motion-tracking system. Surg Endosc.

[29] Azari D, Hu Y, Miller B, Le B, Radwin R (2019). Using Surgeon Hand Motions to Predict Surgical Maneuvers. Hum Factors.

