# Supplementary material for: The use of mixed reality technology for the objective assessment of clinical skills: a validation study
Source: BMC Med Educ. 2022 Aug 23;22:639. doi: 10.1186/s12909-022-03701-3 (PMC9395785; doi:10.1186/s12909-022-03701-3)
Supplement: Supplementary file 1 — Additional file 1: Table S1. History Station (Leg Pain) Mark Scheme. Table S2. Examination Station (Lower Limb) Mark Scheme. Table S3. Procedure Station (Peripheral Cannulation) Mark Scheme. Table S4. Skills Station (Simple Suturing) Mark Scheme. Table S5. History and Assessment (Simulated Patient) Mark Scheme. [file 12909_2022_3701_MOESM1_ESM.pdf]

Table S1 - History Station (Leg Pain) Mark Scheme

	0	1
**Introduction – 5 points**		
Introduction- greeting, own name, full name of patient, role – all to score		
Asks patients D.O.B and takes consent		
Washes Hands using standard Ayliffe technique		
States purpose of interview		
Asks open question early on the interview		
**Presenting Complaint – 15 points**		
Site calf		
Asks about both thigh and buttock		
Duration of symptoms		
When does it occur		
How far can the patient walk		
Asks about rest pain		
Aggravating factors? (walking up hill and into the wind)		
Alleviating factors (when stops walking)		
Asks about Ulcers (previous and current)		
Asks about hypertension (yes)		
History of Diabetes (yes)		
Asks about previous ACS (no)		
History of CVA (no)		
History of back pain (no)		
Previous surgeries (none)		
**Social History – 8 points**		
How does the problem affect daily life		
Alcohol (social drinker 10-14 units a week)		
Smoking (20/day for 20 years = 20 pack years)		
Occupation		
Asks about prescription medication		
Asks about over the counter medication		
Asks how much medication they are taking for the pain		
Asks about medication allergies (none)		
Asks relevant family history		
Relevant review of systems		
**General History Taking Skills – 6 points**		
40-41. **Presentation** – Summarises findings succinctly (done well – score 2, adequate score 1, inadequate score 0)		
42-43. Offers differential diagnoses (one point for any of the following, max two points; critical limb ischaemia, intermittent claudication, acute limb ischaemia, spinal stenosis, diabetic neuropathy)		
44-45. Suggest appropriate investigations (one point for each of the following, maximum two points)		
**Communication Skills – 6 points**		
5-6. Appropriate use of closed questions (done well – score 2, adequate score 1, inadequate score 0)		
7. Avoids using multiple and leading questions		
8. Talks in a language the patient understands		
9-10. Uses empathetic statements and addresses patient concerns (done well – score 2, adequate score 1, inadequate score 0)		

/40

Table S2 - Examination Station (Lower Limb) Mark Scheme

	0	1
**Introduction – 5 points**		
Introduction- greeting, own name, full name of patient, role – all to score		
Asks patients D.O.B and takes consent		
Washes Hands using standard Ayliffe technique		
States purpose of examination		
Asks about current pain or discomfort		
**Physical Examination – 5 points**		
Uses empathetic statements (done well – score 2, adequate score 1, inadequate score 0)		
Appropriately exposes patient		
Offers to assess gait including heel-to-toe walk		
Romberg's test		
**Tone - 4 points**		
Inspection from the end of the bed; mentions at least two of the following (muscle wasting, fasciculations, neurofibromas, scars etc)		
Checks overall tone of legs (both sides)		
Checks for clonus in the ankle (both sides)		
**Power – 5 points**		
Assesses power of hip (flexion and extension +/- abduction & adduction)		
Assesses power of knee (flexion and extension)		
Assesses power of ankle dorsiflexion and plantar flexion		
Assesses power of big toe (flexion and extension)		
Correct technique and comparing both sides		
**Reflexes – 3 points**		
Knee reflexes examined correctly and comparing both sides, with a warning +/- reinforcement (all to score)		
Plantar reflexes examined correctly and comparing both sides, with a warning +/- reinforcement (all to score)		
Co-ordination in the lower limbs (passing foot along shin twice on each side) and comparing both sides		
**Sensation – 6 points**		
Light touch sensation assessed with cotton wool		
Assesses touch sensation in a dermatomal pattern		
Defines clearly the border of the area of sensory deficit		
**Examiner should stop candidate from completing pin prick or temperature sensation and tell them that they are the same for touch. **		
Vibration with 128Hz tuning fork and NOT 256Hz		
Check if the patient can feel vibration sense e.g., using sternum.		
Examination of proprioception using the toes.		
**General Examination Skills – 6 points**		
Examination is performed in an orderly and logical manner (done well – score 2, adequate score 1, inadequate score 0)		
**Presentation** – Summarises findings succinctly (done well – score 2, adequate score 1, inadequate score 0)		
Offers differential diagnoses (one point for any of the following, max two points; critical limb ischaemia, intermittent claudication, spinal stenosis, diabetic neuropathy)		
**Communication Skills – 6 points**		
Explains examination is language the patient understands		
Interacts appropriately with the patient through eye contact and non-verbal skills throughout examination (done well – score 2, adequate score 1, inadequate score 0)		
Gives clear and polite instructions throughout		
Uses empathetic statements (done well – score 2, adequate score 1, inadequate score 0)		

/40

Table S3 - Procedure Station (Peripheral Cannulation) Mark Scheme

	0	1
**Introduction – 5 points**		
Introduction- greeting, own name, full name of patient, role – all to score		
Asks patients D.O.B and checks it against their wristband and/or chart and takes consent		
Washes hands using standard Ayliffe technique		
States purpose of cannulation and explains the procedure		
Asks about needle phobia.		
**Procedure -25 points**		
Wash Hands using appropriate technique		
Cleans tray with 70% alcohol surface wipes		
Assembles the correct equipment in the tray (non-sterile gloves, tourniquet, sterile gauze, skin prep, cannula, sterile IV dressing, sharps bin, protective field underlay, saline flush)		
Puts on disposable plastic apron		
Draws up saline for flushing		
Effort made to remove all air bubbles from the saline flush		
Appropriately positions the patient		
Applies tourniquet 5-10 cm above the site (maximum dwell time 1 min)		
Use other appropriate methods to encourage venous distension as required		
Selects the most suitable site for peripheral cannulation		
Washes Hands again using appropriate technique		
Puts on gloves		
Cleans the skin		
Allows the skin to dry for 30 seconds (do not then repalpate)		
Inserts a cannula through the skin with bevel upwards, at an angle of 15- 45 degrees according to the depth of the vein		
Observe for the first appearance of blood into the flashback chamber of the cannula		
Lower the angle of insertion and advance the cannula a further few millimetres into the vein		
Withdraw the needle *slightly* and observe for a second flashback of blood which will be seen along the shaft of the cannula		
Holding the needle in place, advance cannula off the needle into the vein, until all of the cannula tubing is inserted.		
Removes tourniquet		
Flushes cannula with saline		
Secures cannula using an appropriate technique		
Disposes of all equipment (including sharps) appropriately		
Documents the time and date on the cannula, legibly		
**General Procedural Skills – 4 points**		
Performed the procedure with a good insight into sterilisation technique (done well – score 2, adequate score 1, inadequate score 0)		
Performed the procedure in a logical and fluent manner (done well – score 2, adequate score 1, inadequate score 0)		
**Communication Skills – 6 points**		
Talks in a language the patient understands		
Responds to patients non -verbal clues		
Uses reassuring and empathetic statements (done well – score 2, adequate score 1, inadequate score 0)		
Interacts appropriately with the patient through eye contact and non-verbal skills throughout examination (done well – score 2, adequate score 1, inadequate score 0)		

/40

Table S4 - Skills Station (Simple Suturing) Mark Scheme

	0	1
**Introduction – 5 points**		
Introduction- greeting, own name, full name of patient, role – all to score		
Asks patients D.O.B and checks it against their wristband and/or chart and takes consent		
Washes hands using standard Ayliffe technique		
States purpose of sutures and explains the procedure		
Asks about needle phobia.		
**Procedure - 25 points**		
Clean the trolley and gather equipment.		
Position the patient appropriately		
Prepare the wound area and check that the area has been sufficiently anaesthetised		
Cleans hands a second time		
Open dressing pack and empty out other required equipment on to the sterile field.		
Open non - sterile gloves avoiding contamination of the sterile field.		
Organise layout of items on the sterile field		
Begin to clean tissues with chlorohexidine, starting from the wound edges in an outwards motion away from the wound.		
Apply a sterile paper drape with an appropriate window cut into it.		
Map out where you plan to insert your sutures, noting the position of wound edges and skin folds.		
Insert the first suture in the middle of the wound and then continue dividing into equal sections – approximately 5-10mm apart.		
Grasp the needle two thirds of the way from the needle's point with the needle holder.		
Holding the skin with the forceps, pierce the skin at a 90° angle not closer than 5mm from the wound edge, following the curvature line of the needle as it passes through the tissue, into the middle of the wound.		
Remove the needle and remount it in the needle holder before taking a corresponding bite on the other side of the wound. Do not touch with fingers to avoid needle stick injury. Hold the needle with forceps while repositioning it in the needle holder.		
Pull the suture through until approximately 15cm remains.		
Ties with a reef knot (instrument tie), ensuring that all knots end up on the same side		
Cuts the end of the suture to an appropriate length e.g. 5-10mm.		
Clean the wound and apply gentle pressure to ensure bleeding ceases.		
Counts three sutures before applying a non-adherent dressing		
Dispose of equipment safely (including sharps) and appropriately and remove gloves.		
Gives simple post care advice to patient		
**Quality of sutures – 3 points**		
Sutures are suitable tensioned		
Sutures are suitable spaced		
Each knot contains sufficient ties		
**General Skills – 4 points**		
Performed the procedure with a good insight into sterilisation technique (done well – score 2, adequate score 1, inadequate score 0)		
Performed the procedure in a logical and fluent manner (done well – score 2, adequate score 1, inadequate score 0)		
**Communication Skills – 6 points**		
Talks in a language the patient understands		
Responds to patients non -verbal clues		
Uses reassuring and empathetic statements (done well – score 2, adequate score 1, inadequate score 0)		
Interacts appropriately with the patient through eye contact and non-verbal skills throughout examination (done well – score 2, adequate score 1, inadequate score 0)		

/40

Table S5 - History and Assessment (Simulated Patient) Mark Scheme

**General Inspection – 3 points**	
Excellent (3 points)<br>Provides a through and succinct summary of the patient including all of the following: age, state of breathlessness, cough, additional breathing sounds. Student comments on the absence of additional support around the bed and on the vital signs.<br>Good (2 points)<br>The student summarises the patient with at least 3 out of 5 of the following: age, state of breathlessness, cough, additional breathing sounds. Student comments on the absence of additional support around the bed and on the vital signs.<br>Adequate (1 point)<br>The student summarises the patient with at least 2 out of 5 of the following: age, state of breathlessness, cough, additional breathing sounds.<br>Poor (0 points)<br>The student summarises the patient with less than 2 out of 5 of the following: age, state of breathlessness, cough, additional breathing sounds.	
**Management of a deteriorating patient, re-evaluation and management- 25 points**	
Excellent (25 points)<br>Student suggests **all** of the following initial investigations: FBC, ABG, CXR, Ferritin, CRP, IL-6 and D-dimer. When prompted – what's changed, the student comments on the patient's appearance including both increasing breathlessness and increasing respiratory effort.<br>Student re-evaluates the patient at every step and makes changes to the management accordingly. Student correctly identifies the need for additional oxygen support. They suggest a nasal cannula or non -rebreather mask within the first two stages. Before stage three they suggest adding CPAP or more invasive ventilation support.<br>Very Good (20 points)<br>Student suggests 4 out of 7 of the following initial investigations: FBC, ABG, CXR, Ferritin, CRP, IL-6 and D-dimer. When prompted – what's changed, the student comments on the patient's appearance including both increasing breathlessness and increasing respiratory effort.<br>Student re-evaluates the patient at every step and makes changes to the management accordingly. Student correctly identifies the need for additional oxygen support. They suggest a nasal cannula or non -rebreather mask within the first two stages.<br>Good (15 points)<br>Student suggests 3 out of 7 of the following initial investigations: FBC, ABG, CXR, Ferritin, CRP, IL-6 and D-dimer. When prompted – what's changed, the student comments on the patient's appearance mentioning either increasing breathlessness or increasing respiratory effort.<br>Student re-evaluates the patient at every step and makes changes to the management accordingly. Student correctly identifies the need for additional oxygen support. They suggest a nasal cannula or non -rebreather mask within the first two stages.<br>Adequate (10 points)<br>Student suggests 2 out of 7 of the following initial investigations: FBC, ABG, CXR, Ferritin, CRP, IL-6 and D-dimer. When prompted – what's changed, the student comments on the patient's appearance mentioning either increasing breathlessness and increasing respiratory effort.<br>Student re-evaluates the patient at every step and makes changes to the management accordingly.<br>Poor (5 points)<br>Student suggests 1 out of 7 of the following initial investigations: FBC, ABG, CXR, Ferritin, CRP, IL-6 and D-dimer.<br>Student re-evaluates the patient at every step and makes changes to the management accordingly.	
**Interpretation of vital signs and MEWS calculation – 5 points**	

Excellent (5 points)<br>Student is aware of normal ranges of vital signs and comments on at least 2 figures that are out of the normal range. Student can identify concerning vital signs at every stage. Student can identify the continued fall of oxygen saturation despite increasing support.<br>Very Good (4 points)<br>Student is aware of the majority of normal ranges of vital signs and comments on one figure that are out of the normal range. Student can identify concerning vital signs at every stage. Student can identify the continued fall of oxygen saturation despite increasing support.<br>Good (3 points)<br>Student can identify concerning vital signs at every stage. Student can identify the continued fall of oxygen saturation despite increasing support.<br>Adequate (2 points)<br>Student can identify some concerning features but does not comment on the continued fall of oxygen saturation despite increasing support.<br>Poor (1 points)<br>The student fails to identify vital signs which are out of the normal ranges and does not comment on the continued fall of oxygen saturation despite increasing support.	
**SBAR– 4 points**	
Excellent (4 points)<br>Student logically and succinctly presents the patient in a SBAR format including all features, situation, background, assessment and recommendation.<br>Very Good (3 points)<br>Student presents the patient in a SBAR format including all features, situation, background, assessment and recommendation including some additional details.<br>Good (2 points)<br>Student includes 3 out of 4 of the components in their summary: situation, background, assessment and recommendation.<br>Adequate (1 points)<br>Student includes 1 out of 4 of the components in their summary: situation, background, assessment and recommendation.<br>Poor (0 points)<br>Student includes 0 out of 4 of the components in their summary: situation, background, assessment and recommendation.	
**Communication Skills – 6 points**	
Excellent (6 points)<br>Student explains their management clearly and succinctly with rationale for their decision making. The student listens to prompts from the examiner and responds appropriately.<br>Very Good (4 points)<br>Student explains their management clearly and succinctly but without rationale for their decision making. The student listens to prompts from the examiner and responds appropriately.<br>Good (3 points)<br>Student explains their management clearly but with repetition and without rationale for their decision making. The student listens to prompts from the examiner and responds appropriately.<br>Adequate (2 points)<br>Student explains their management but with repetition and without rationale for their decision making.<br>Poor (0-1 points)<br>Communication is unclear and the student does not respond reactively to prompts from the examiner.	
	**/43**
